# Optimization Method for the Synergistic Control of DRIE Process Parameters on Sidewall Steepness and Aspect Ratio

**DOI:** 10.3390/mi17010013

**Published:** 2025-12-23

**Authors:** Dandan Wang, Cheng Lei, Pengfei Ji, Zhiqiang Li, Renzhi Yuan, Jiangang Yu, Ting Liang, Zong Yao, Jialong Li

**Affiliations:** 1 State Key Laboratory of Widegap Semiconductor Optoelectronic Materials and Technologies, North University of China, Taiyuan 030051, China; s202319038@st.nuc.edu.cn (D.W.); mnfc@nuc.edu.cn (P.J.); 20240101@st.nuc.edu.cn (Z.L.); liangtingnuc@nuc.edu.cn (T.L.); 2 Beijing NAURA Microelectronics Equipment Co., Ltd., Beijing 100176, China; yuanrenzhi@naura.com; 3 North Automatic Control Technology Institute, Taiyuan 030006, China; yaozong126@sina.com (Z.Y.); ljl18893953916@163.com (J.L.)

**Keywords:** sidewall, single-step time, RF power, top-bottom line width, passivation-etch time ratio

## Abstract

Deep Reactive Ion Etching (DRIE), as a key process in silicon micromachining, remains constrained in high-precision applications by sidewall angle deviation and aspect ratio limitations. This study systematically investigates the mapping relationship between process parameters and etching morphology, focusing on the following aspects: the influence mechanism of C_4_F_8_ passivation time and bottom RF power on sidewall perpendicularity; and the effect patterns of etch cycle count, single-step time, and bottom RF power on aspect ratio and top–bottom line width (CD) difference. The findings reveal that dynamic adjustment of bottom RF power significantly influences sidewall angle: incremental adjustment tends to cause sharp angles (decreased angular precision), while decremental adjustment tends to form obtuse angles. Simply increasing the cycle count leads to a bottleneck in etch depth growth. Combining incremental bottom RF power adjustment can overcome depth limitations but induces axial variation in aperture dimensions. Optimizing the passivation-to-etch time ratio effectively controls etch morphology characteristics. This study achieved an etch depth of 112.2 μm for a 5 μm wide trench with an overall aperture size difference of 0.279 μm, providing a theoretical basis and practical guidance for parameter optimization in DRIE processes for high-precision silicon structure fabrication.

## 1. Introduction

As a core technology comprising etching and passivation cycles, the Bosch process holds a pivotal position in the fabrication of high-aspect-ratio silicon microstructures [1,2,3]. However, this process faces dual challenges in practice. On one hand, achieving anisotropic etching with vertical sidewall angles while maintaining consistent trench opening dimensions remains an industry-wide challenge [4,5]. On the other hand, micro-scale pattern etching is constrained by etch depth (or aspect ratio): as the number of etch cycles increases, reactive ions and gases struggle to effectively diffuse into confined etch regions, directly limiting process performance improvements [6].

The root cause of these issues lies in the complexity of the process system. Within each etching/passivation cycle, multiple parameters—including chamber pressure, etching/passivation gas flow rates, the etching-to-passivation time ratio, substrate temperature, center RF power, and bottom RF power—must be precisely controlled to establish a balanced process system [7,8,9,10]. Only through such precise control can effective regulation of etch dimensions and aspect ratio be achieved, while simultaneously ensuring consistency in silicon trench aperture size and verticality of sidewalls [11,12].

To overcome these limitations, research has focused on optimizing DRIE process parameters for micro-scale patterns, aiming to improve etch profiles and enhance etch depth [13,14,15]. For example, research has achieved TSV hole etching with a diameter of 50 μm and a depth of 300 μm [16]. Exploration of Bosch processes with aspect ratios ranging from 3:1 to 7.5:1 revealed that controlling anisotropy and roughness becomes exceptionally challenging when the aspect ratio exceeds 15 [17]. In studies of 15 μm diameter hole arrays, aspect ratios as high as 26 have been achieved [18]. However, investigations into smaller dimensions reveal that the aspect ratio of optimized 4 μm diameter holes can reach 12.5 [19,20]; their cross-sectional area decreases with increasing aspect ratio, resulting in a bottom dimension less than half that of the top.

It is evident that for deep etching of microstructures based on Bosch processes, particularly in scenarios involving small apertures, a comprehensive understanding of the deep silicon etching mechanism under conditions of vertical sidewall angle and trench aperture size consistency holds critical practical significance.

Accordingly, this study proposes an optimized method for small-aperture etching, focusing on improving aspect ratio and uniformity of trench aperture size. Through systematic investigation of the effects of Bosch process cycles and bottom radiofrequency power on etch depth, anisotropy, sidewall angle, and aperture size uniformity, an experimental analysis-based silicon etching equilibrium model is established. This provides a universal technical solution for deep etching in the fabrication of silicon microstructures.

## 2. Materials and Methods

**Figure 1:**
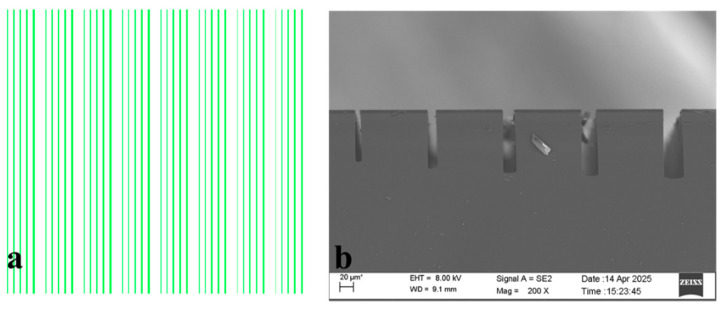
(**a**) Mask pattern: 5–25 × 5000 μm, with 100 μm spacing between patterns and 200 μm array pitch. (**b**) Scanning electron microscope image of the standard process.

Spin-coat 4620 photoresist onto 4-inch n-type silicon wafers with a crystal orientation <100> and a resistivity of 1–10 Ω·cm, achieving a film thickness of 6 μm. Form the etching pattern via UV exposure, with feature dimensions of 5–25 × 5000 μm, as shown in Figure 1a. Subsequently, etch the samples using a three-step Bosch process. First, a base recipe specified in Table 1 was employed, with parameters including etch cycle count, bottom RF power, C4F8 introduction time and flow rate, and SF6 introduction time. Standard process results are shown in Figure 1b. Using these parameters for etching, the etch depth-to-width ratio was enhanced by varying the etch cycle count, C_4_F_8_ introduction time, C_4_F_8_ gas flow rate, SF6 introduction time, and bottom RF power to observe their effects [21].

Each set of experimental parameters was etched using three 4-inch silicon wafers to ensure repeatability. The measured aperture size represents the average of three measurements.

## 3. Results

The optimized Bosch process comprises three steps: first, passivation with C_4_F_8_ [22,23,24,25]; second, opening the passivation layer via high-energy bombardment with SF_6_; and third, etching through chemical reaction between SF_6_ and Si. During the first deposition step, fluorocarbon compounds form on the bottom and sidewalls to provide protection. In the second step, high RF power at the bottom creates strong ion traction, physically bombarding the passivation layer to open it for chemical etching in the third step. Chemical etching can be categorized into the following two types: spontaneous isotropic etching of F* groups and ion-enhanced F* group etching. The four process parameters studied in this paper are divided into the following two parts: the number of etching cycles, the introduction time and flow rate of C_4_F_8_, and the introduction time of SF_6_, which influence the final etching morphology through chemical reactions. Meanwhile, bottom RF power primarily affects etching morphology through physical bombardment. The etching parameters are shown in Table 2.

**Figure 2:**
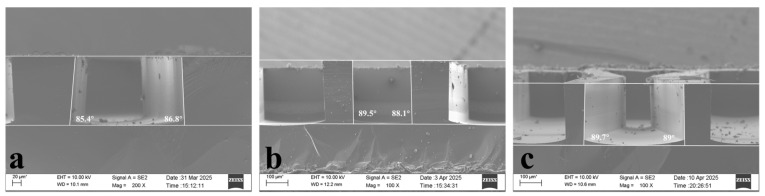
(**a**–**c**) are scanning electron microscope images corresponding to bottom radiofrequency power levels of 100–120 W, 100–100 W, and 100–80 W, respectively.

The three-step Bosch process allows adjustment of the bottom RF power at the start and end stages, enabling a linear increase or decrease in bottom RF power as etch depth increases. Due to the excessively small size of the 5 μm aperture, direct measurement of angle error is imprecise. Therefore, etching studies were first conducted on patterns with larger apertures. Three sets of experiments were performed with bottom RF powers of 100–120/100–100/100–80, respectively. The results are shown in Figure 2. The sidewall angles were 86.6°/88.8°/89.35°, respectively.

Directly correlated with etch depth is the number of etch cycles. As the number of etch cycles increases, the etch depth correspondingly increases. Therefore, three sets of experiments were conducted with the number of etch cycles as the variable.

**Figure 3:**
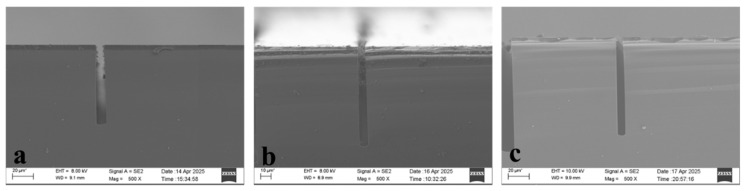
(**a**–**c**) show SEM images after 100, 150, and 180 etching cycles, respectively.

After 100 etching cycles, the etch depth of a 5 μm wide trench reached 77.49 μm, as shown in Figure 3a. When the number of etching cycles was increased to 150, the etch depth reached 96.24 μm, as shown in Figure 3b. Further increasing the cycles to 180 yielded an etch depth of 95.57 μm, as shown in Figure 3c, which was close to the depth achieved at 150 cycles. This demonstrates that simply increasing the number of etching cycles does not lead to further increases in etch depth. On one hand, as etch depth increases, fluorine radicals must traverse longer paths to reach the trench bottom, frequently colliding with passivated layers on the sidewalls along the way, thereby reducing the effective fluorine atom concentration. On the other hand, the escape path for etch products lengthens in high-aspect-ratio structures, forming local concentration gradients that inhibit the forward progression of the chemical reaction between fluorine and silicon [26].

To enable gas penetration into deeper etching regions, we devised the following two approaches: one involves increasing the bottom RF power to enhance the bottom’s attraction to ions, allowing them to reach the etching bottom and continue downward etching; the other extends the etching gas introduction time to increase ion concentration, thereby promoting downward etching. Consequently, the following experiments were conducted.

As etch depth increased, both etch gas flow time and bottom RF power were ramped up. When the chemical etch time was adjusted from a constant 3 s to 3.5 s as the number of etch cycles increased, and the bottom RF power was increased from 100 W to 120 W over the same number of cycles, the etch depth reached 114.3 μm.

High bottom RF power accelerates ions to high kinetic energy. When these ions bombard the bottom of the trench, a portion rebounds due to elastic collision with the silicon surface. The rebounded ions have a lateral velocity component, which bombards the sidewall passivation layer. As etch depth increases, the trench aspect ratio increases, and the probability of ion rebound increases (ions are confined by the trench sidewalls and undergo multiple collisions). This leads to cumulative damage to the sidewall passivation layer from top to bottom, with the bottom passivation layer being the most severely damaged. The damaged passivation layer cannot effectively block F* radicals (the main etching species derived from SF_6_ dissociation). F* radicals react with silicon in an isotropic manner. The more severely damaged passivation layer at the bottom of the trench allows more F* radicals to react with sidewall silicon, resulting in greater lateral etching at the bottom than at the top. This cumulative lateral etching causes the aperture size to increase with depth [27]. Additionally, the transport of charged particles within narrow trench structures is constrained by the confined space, leading to significant charge accumulation that increases with rising aspect ratio. High-energy ions induced by bottom RF power are predominantly positively charged. As the aspect ratio increases, these positive ions become confined within deep trenches, hindering their diffusion. Meanwhile, electrons with higher mobility are more likely to escape or be captured by the passivation layer on the sidewalls. This results in a net positive charge accumulation at the trench bottom, forming a localized electric field extending from the trench floor to the sidewalls. Under this electric field, subsequent incident positive ions experience lateral repulsive forces. These forces deflect their trajectories toward the sidewalls superimposed on the lateral velocity component of the rebounding ions, further intensifying the bombardment of the bottom sidewall passivation layer. Consequently, lateral etching causes the aperture to increase with depth [28].

**Figure 4:**
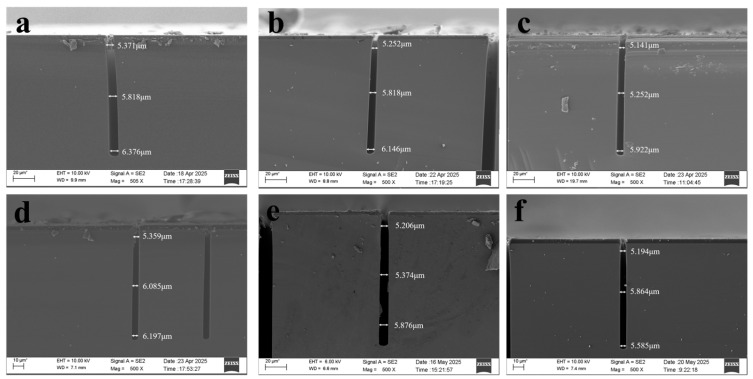
SEM images of etching results. (**a**,**b**) SEM results from the first experimental group; (**c**) SEM results from the second experimental group; (**d**,**e**) SEM results from the third experimental group; and (**f**) SEM results from the fourth experimental group.

**Figure 5:**
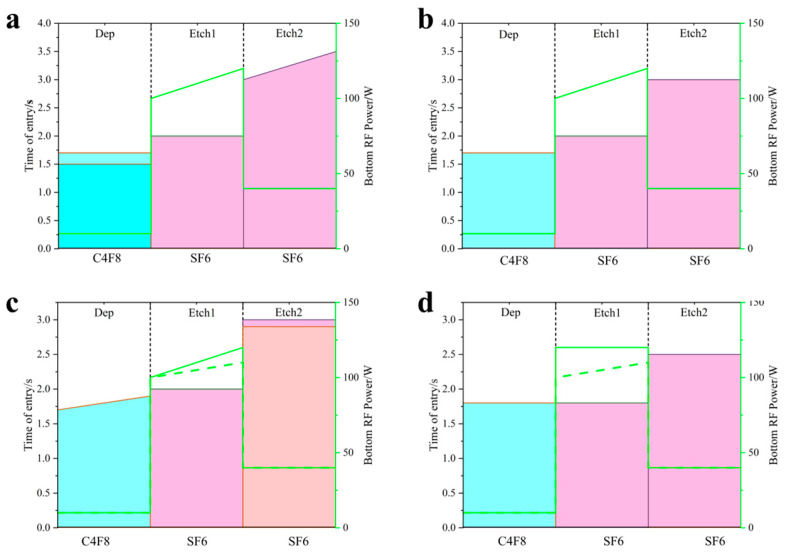
Etching parameter diagram. (**a**) Blue bars represent C_4_F_8_ introduction time (lighter blue indicates the second experimental group, identical to the first group except for this variable); pink bars denote SF_6_ introduction time for steps two and three; and the green dashed line shows bottom RF power level. (**b**) Blue bars represent C_4_F_8_ introduction time; pink bars represent SF_6_ introduction time in the second and third steps; and the green dashed line indicates bottom RF power level. (**c**) Blue bars represent C_4_F_8_ introduction time; pink bars represent SF6 introduction time in the second and third steps (orange indicates variables for the second experimental group); and the green dashed line indicates bottom RF power level (dashed line indicates variables for the second experimental group). (**d**) Blue bars represent C_4_F_8_ introduction time; pink bars indicate SF_6_ introduction time for the second and third steps; and the green dashed line shows bottom RF power level (dashed line denotes the second set of experimental variables).

Specifically, the opening dimensions at the top, middle, and bottom of the pattern exhibit variations, with a maximum difference of 1.005 μm (between the top and bottom)as shown in Figure 4a. To mitigate this dimensional variation, analysis indicated insufficient sidewall passivation as a potential cause. Consequently, the passivation gas exposure time was appropriately extended from 1.5 s to 1.7 s, as shown in Figure 5a, reducing the maximum opening size difference to 0.894 μm, as shown in Figure 4b.

Increasing the passivation gas exposure time yields a slight improvement in opening size variation. To enhance sidewall passivation, one can either extend the passivation gas exposure time or increase the passivation gas flow rate. Both approaches enhance sidewall passivation and reduce opening size variation. Alternatively, decreasing etch time lowers etch ion concentration, thereby reducing consumption of sidewall passivation and improving opening size variation. To further minimize opening size variation, the edge passivation gas flow rate was increased from 0 sccm to 50 sccm. The etch time was reduced back to the original 3 s, as shown in Figure 5b, resulting in a reduced opening size difference of 0.781 μm, as shown in Figure 4c.

The improvements to the above formulation had little effect on dimensional variation. Further analysis revealed that since bottom RF power increases with etch depth, extending passivation time also increases with etch depth. An alternative approach involved further reducing etch gas time while diminishing the increase in bottom RF power with etch depth. This reduced ion traction, decreased sputtering energy upon bottom impact, and consequently minimized consumption of sidewall passivation. Based on this, two sets of experiments were conducted. One set extended the passivation gas introduction time from 1.7 s to 1.9 s as the number of etching cycles increased, as shown in Figure 5c, reducing the aperture size difference to 0.838 μm, as shown in Figure 4d. The other group further reduced the etching gas time to 2.9 s and decreased the bottom RF power from 100–120 to 100–110,as shown in Figure 5d, narrowing the aperture size difference to 0.67 μm, as shown in Figure 4e.

Following the aforementioned formulation adjustments, the variation in aperture size difference remained insignificant. Therefore, the formulation underwent comprehensive modification. The passivation time was uniformly set to 1.8 s regardless of etch depth. Similarly, the second-step passivation time was fixed at 1.8 s irrespective of etch depth. This reduction in duration decreased ion concentration, lowered bombardment intensity, and minimized consumption of sidewall passivation. The third-step chemical etching time was adjusted to 2.5 s, lowering the ion concentration and reducing chemical etching. The bottom RF power was set to 100–110, with edge passivation gas flow at 50 sccm. The etching results showed an aperture size difference of 0.67 μm, exhibiting faster etching in the middle region and smaller apertures at the top and bottom (bowing effect), as shown in Figure 4f.

In the Bosch process, plasma is generated in the reaction chamber and diffuses into the trench. Due to the confinement effect of the trench sidewalls, the plasma density in the middle region of the trench is higher than that at the top and bottom. Higher plasma density means more F* radicals and energetic ions, leading to a higher etching rate in the middle. The passivation layer (fluorocarbon film) is deposited by C_4_F_8_ plasma. The top of the trench is directly exposed to plasma, resulting in a thicker passivation layer. The bottom of the trench has a lower plasma density due to diffusion limitations, leading to a thinner passivation layer, but this is being compensated by the rebound ion bombardment. The middle region has a moderate plasma density and reduced ion bombardment, resulting in a thinner passivation layer than the top but thicker than that at the bottom. This non-uniform passivation layer thickness causes the middle region to be more susceptible to lateral etching, further exacerbating the bowing effect.

**Figure 6:**
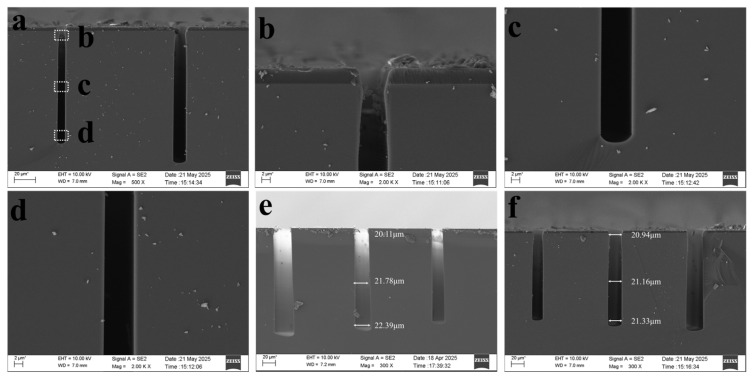
SEM images of etching results. (**a**) Overall etching morphology; (**b**) top enlarged morphology; (**c**) middle enlarged morphology; (**d**) bottom enlarged morphology; (**e**) shows the initial etching result at 20 μm; and (**f**) shows the final etching result at 20 μm.

**Figure 7:**
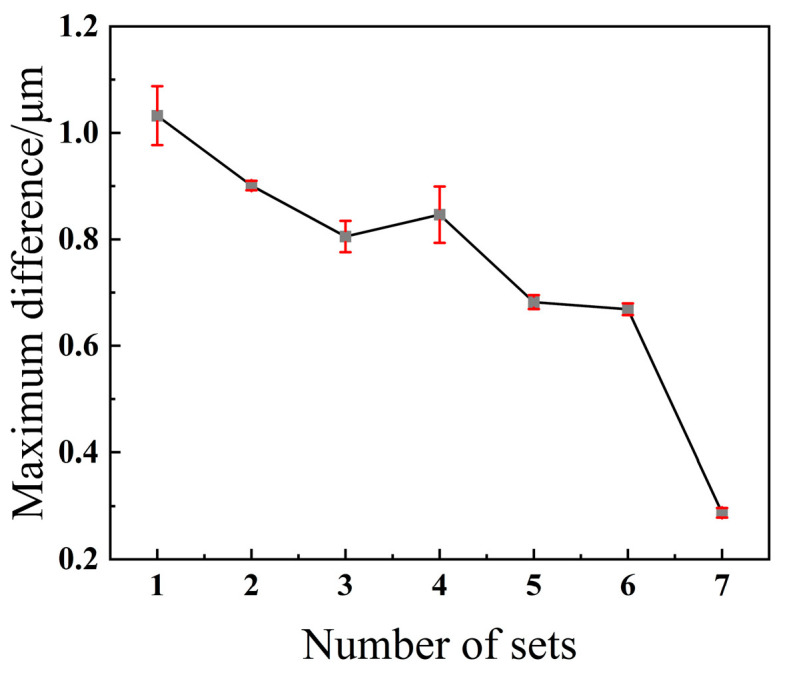
Data were obtained from three independent replicates of each experimental group.

Sputtering on the sidewalls facilitates simple and convenient operation across various etch depths by eliminating variables that change with etch cycle count. Therefore, based on the aforementioned recipe, the bottom RF power was adjusted to 120 W. The resulting etch aperture size variation was 0.279 μm, as shown in Figure 6a–d. Each set of experiments was repeated three times, and the measured opening size differences were calculated. The error plot is shown in Figure 7. The overall recipe contains no variables that change with cycle count, and the aperture size variation also reached 0.279 μm. A comparative analysis was conducted on patterns with an aperture size of 20 μm using the same etching parameters as above. Only the initial and final results were compared, and the dimensional difference between the initial and final etching results was 0.39 μm, as shown in Figure 6e,f.

**Figure 8:**
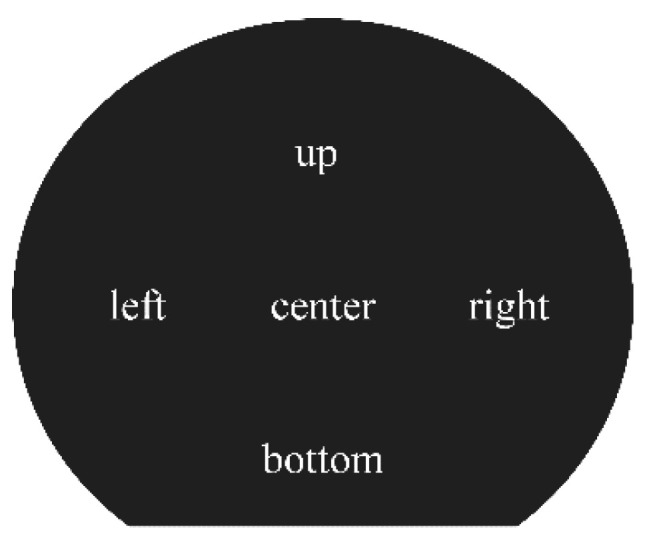
Four inch wafer test position.

Finally, a three-step Bosch process was employed to etch 4-inch silicon wafers using optimized parameters. The uniformity of bottom corner height differences and etch depth uniformity were measured at the following five points: top, center, bottom, left, and right. The measurement locations are shown in Figure 8. The measured etch depth and aperture size difference data for the five points are presented in Table 3. The etch depth uniformity was 3.09%. The uniformity formula is as follows:

(1) $$Uniformity=\frac{D_{\max}-D_{min}}{2\times D_{average}}$$

**Figure 9:**
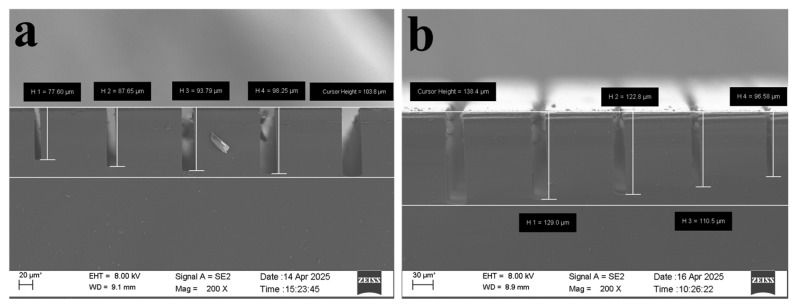
SEM images of etching results: (**a**) 150 etch cycles and (**b**) increasing etch gas time and bottom RF power etch result.

Based on the above results, it can also be concluded that increasing etching gas time and bottom RF power causes the etch rate of large-aperture patterns to increase faster than that of small-aperture patterns. This leads to a greater difference in etch depth between patterns with different aperture sizes on the same wafer, as shown in Figure 9, where the etch depth difference increased from 26.2 μm to 41.82 μm.

## 4. Discussion

This study investigates the plasma etching process for 5 μm wide trench structures, with the core objective of enhancing the etch depth-to-width ratio while reducing overall opening size variation (i.e., dimensional deviation between the top, middle, and bottom openings of the trench). It systematically summarizes the process optimization strategies to achieve this goal. The results demonstrate that precise control of key Bosch process parameters—including single-step deposition time, single-step etching time, and bottom RF power—significantly improves trench D/A ratio and effectively reduces overall opening size variation, successfully achieving the predetermined research objectives. The core mechanism of this process control strategy is as follows: Optimizing bottom RF power enhances the ion capture capability at the trench bottom, alleviating the shortage of etchant supply at the bottom caused by gas replacement lag and addressing the critical bottleneck of “insufficient bottom etching drive” during DBR enhancement. Simultaneously, it reduces directional deviation during ion sputtering, minimizing non-uniform etching caused by ion reflection. Simultaneously, rational matching of single-step deposition time enables stable protective layer formation on trench sidewalls, preventing excessive sidewall etching. The synergistic effect of bottom RF power and single-step deposition time further suppresses expansion of aperture size deviation, ultimately achieving precise control over trench morphology.

The optimization scheme proposed in this study provides a critical theoretical foundation and directly implementable technical solution for developing plasma etching processes for “high-precision trench structures” in microelectromechanical system (MEMS) device manufacturing. It holds clear practical value and promising application prospects for advancing the manufacturing precision of core MEMS device structures, such as microfluidic channels and sensor electrode trenches.

## Figures and Tables

**Table 1:** Basic formulation.

Parameters	Cycles	Time/s	Center C_4_F_8_/sccm	Edge C_4_F_8_/sccm	Center SF_6_/sccm	Edge SF_6_/sccm	LFPower/W
Dep	100	1.5	180	0	0	0	10
Etch1	100	2	0	0	300	300	100
Etch2	100	3	0	0	300	300	40

**Table 2:** Etching parameter experiments.

	Parameters	Cycles	Time (s)	Center C_4_F_8_/sccm	Edge C_4_F_8_/sccm	LFPower
A	Dep	100→150→180	1.5	180	0	10
	Etch1		2	0	0	100
	Etch2		3	0	0	40
B	Dep	180	1.5/1.5→1.7	180	0	10
	Etch1		2	0	0	100–120/100–110
	Etch2		3–3.5/3–3.2	0	0	40
C	Dep	180	1.7/1.7–1.9	180	50	10
	Etch1		2	0	0	100–110→100–120
	Etch2		2.9→3	0	0	40
D	Dep	200	1.8	180	50	10
	Etch1		1.8	0	0	100–100→120
	Etch2		2.5	0	0	40

**Table 3:** Etching results data.

Position	Etch Depth/μm	Opening Size Difference/μm
center	106.662	0.279
up	106.1	0.279
bottom	108.3	0.335
left	109.427	0.340
right	108.3	0.340

## Data Availability

The original contributions presented in this study are included in the article. Further inquiries can be directed to the corresponding author.
